# SUMO3 Modification Accelerates the Aggregation of ALS-Linked SOD1 Mutants

**DOI:** 10.1371/journal.pone.0101080

**Published:** 2014-06-27

**Authors:** Takako Niikura, Yoshiko Kita, Yoichiro Abe

**Affiliations:** 1 Department of Information and Communication Sciences, Faculty of Science and Technology, Sophia University, Tokyo, Japan; 2 Department of Pharmacology, Keio University School of Medicine, Tokyo, Japan; University of Florida, United States of America

## Abstract

Mutations in superoxide dismutase 1 (SOD1) are a major cause of familial amyotrophic lateral sclerosis (ALS), whereby the mutant proteins misfold and aggregate to form intracellular inclusions. We report that both small ubiquitin-like modifier (SUMO) 1 and SUMO2/3 modify ALS-linked SOD1 mutant proteins at lysine 75 in a motoneuronal cell line, the cell type affected in ALS. In these cells, SUMO1 modification occurred on both lysine 75 and lysine 9 of SOD1, and modification of ALS-linked SOD1 mutant proteins by SUMO3, rather than by SUMO1, significantly increased the stability of the proteins and accelerated intracellular aggregate formation. These findings suggest the contribution of sumoylation, particularly by SUMO3, to the protein aggregation process underlying the pathogenesis of ALS.

## Introduction

Amyotrophic lateral sclerosis (ALS) is a progressive neurodegenerative disorder that causes the selective loss of motor neurons leading to paralysis and ultimately death within 2–5 years. Although most ALS cases are sporadic, approximately 10% of familial ALS cases are inherited in an autosomal dominant manner. Mutations in superoxide dismutase 1 (SOD1) are the second most common cause of familial ALS (FALS) after C9ORF72 [1], [2]. SOD1 mutants have been widely used for *in vitro* and *in vivo* models to investigate the pathomechanisms of ALS [3], [4].

Mice or rats overexpressing FALS-linked SOD1 mutants develop a human ALS-like phenotype that involves motor neuron degeneration. FALS-linked mutant SOD1 proteins misfold and aggregate into intracellular inclusions both *in vitro* and *in vivo* [5], and it is generally accepted that the propensity for aggregation is associated with the pathobiology of SOD1 mutants [4]–[6]. The aggregation of disease-specific proteins is implicated in the pathogenesis of other neurodegenerative disorders, such as amyloid β in Alzheimer’s disease and α-synuclein in Parkinson’s disease. Therefore, elucidating the process of aggregate formation is important for understanding the pathomechanisms of ALS and other neurodegenerative disorders in which pathological intraneuronal inclusions develop.

Sumoylation, a post-translational protein modification, involves the covalent attachment of small ubiquitin-like modifier (SUMO) proteins to target proteins. Although SUMO is typically conjugated to a specific lysine (Lys) residue within the consensus motif ψKxE/D (where ψ is an aliphatic amino acid) in the target protein, sumoylation can occur at Lys residues outside of the consensus, and not all the consensus Lys residues are sumoylated [7]. The mechanism of the conjugation of SUMO to its target protein is similar to that of ubiquitination. Sumoylation requires a cascade with three enzymes, E1 activating, E2 conjugating, and E3 ligase enzymes; however, unlike ubiquitin, at least three SUMO family proteins, SUMO1, -2, and -3, exist [8], [9]. SUMO1 only displays 45% sequence identity with SUMO2 and SUMO3, whereas SUMO2 and SUMO3 are 87% identical [10]. SUMO1 and SUMO2/3 serve distinct functions by targeting different proteins [11], [12], though some substrates can be modified by both SUMO1 and SUMO2/3. For instance, RanGAP1, α-synuclein, and tau are predominantly modified by SUMO1 and to a lesser extent by SUMO2/3 [13], [14]. In addition, SUMO2/3 but not SUMO1 can form polymeric chains [15], and the de-sumoylating enzymes, sentrin/SUMO-specific proteases (SENPs) 3, 5, 6, and 7, preferentially act on SUMO2/3 versus SUMO1 [16]. It is therefore postulated that SUMO1 and SUMO2/3 can cause different functional consequences on the same substrate. Sumoylated proteins have been implicated in the pathogenesis of several neurodegenerative disorders, such as huntingtin in Huntington’s disease, tau and amyloid precursor protein in Alzheimer’s disease, and α-synuclein and DJ-1 in Parkinson’s disease [8], [17]. In ALS model mice, C-terminal fragment of excitatory amino acid transporter 2 (EAAT2) cleaved by caspase 3 is modified by SUMO1 and accumulates in spinal cord astrocytes [18]. The astrocytic expression of sumoylated EAAT2 fragment induces cytotoxicity in NSC34 cells and primary motor neurons [19]. These findings provide evidence for the involvement of sumoylation in ALS pathogenesis. Since SUMOs are expressed in spinal cord neurons [20], [21], it is assumed that sumoylation may have a pathological role in motor neurons as well as astrocytes. Fei *et al.* reported that the human SOD1 protein is sumoylated and stabilized by SUMO1, suggesting that sumoylation is linked to SOD1 aggregation [22]. However, the detailed mechanisms of the relationship to ALS pathogenesis remain unclear. In the present study, we investigated the effect of sumoylation on ALS-linked mutant SOD1 proteins in a motor neuron cell line and found that SOD1 is sumoylated not only by SUMO1 but also by SUMO2/3, suggesting a role for SUMO2/3 in the pathogenesis of ALS.

## Results

### SUMO1 modification of SOD1 at both Lys9 and Lys75 in motoneuronal NSC34 cells

**Figure 1:**
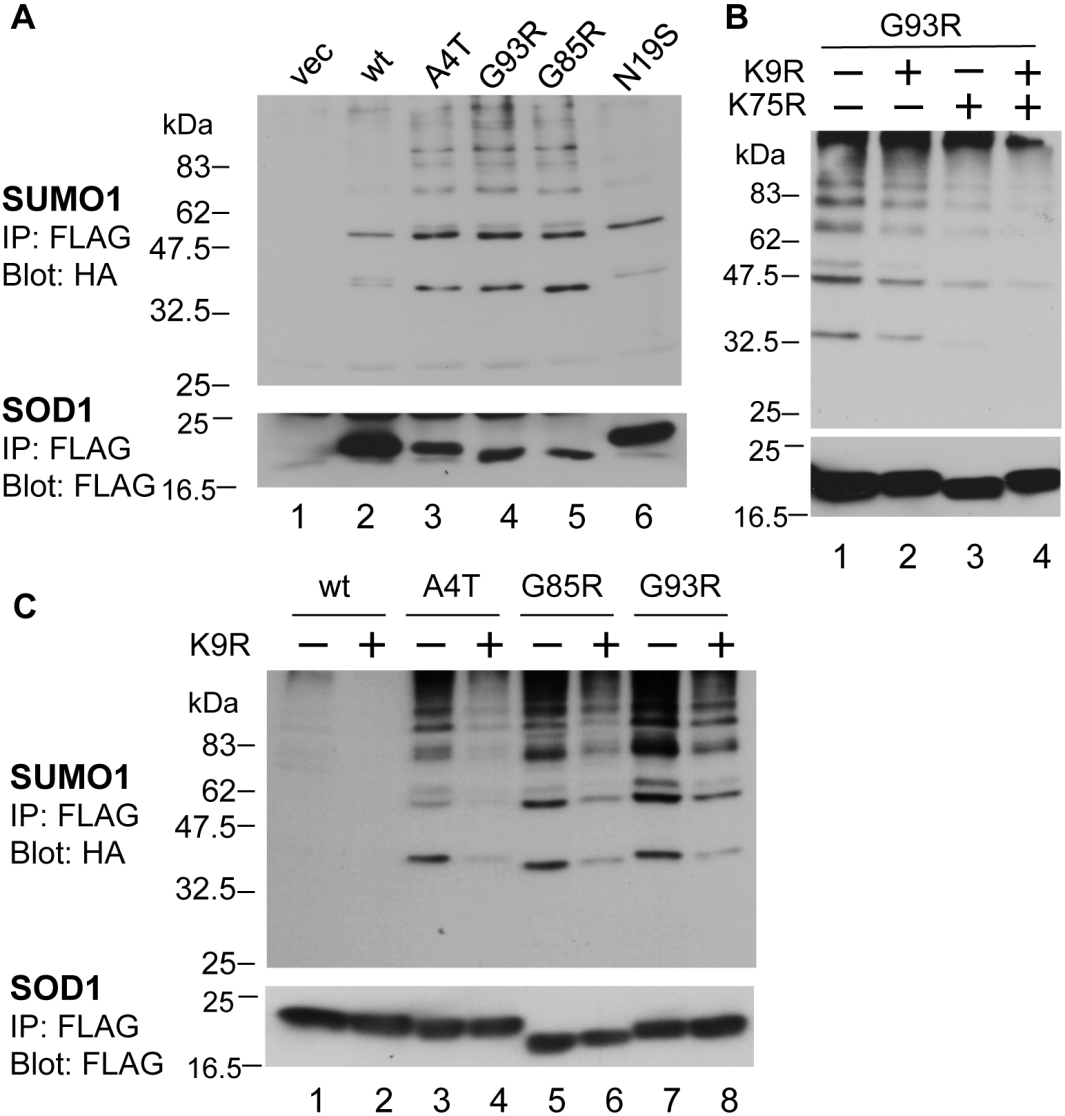
Familial ALS-linked SOD1 mutants are modified by SUMO1 at Lys9 and Lys75. NSC34 (**A, B**) or HEK293 cells (**C**) were cotransfected with plasmids expressing FLAG-tagged wild-type (wt), mutant SOD1, or empty vector (vec) and HA-tagged SUMO1 and myc-tagged Ubc9. The cell lysates were immunoprecipitated with anti-FLAG M2 antibody, followed by immunoblotting with anti-HA antibody (upper panel) and anti-FLAG antibody (lower panel). **A.** SUMO1 modification of SOD1 proteins in NSC34 cells. All SOD1 proteins, wt, familial ALS-linked mutants (A4T, G93R, and G85R), and sporadic ALS mutant (N19S), were modified by SUMO1. A representative immunoblot result from three independent experiments is shown. **B.** Sumoylation of G93R-SOD1 by SUMO1 was decreased by both K9R and K75R mutations. The presence (+) or absence (−) of mutations at K9 and K75 in each construct is indicated above the lane. A representative immunoblot result from three independent experiments is shown. **C.** Sumoylation of SOD1 proteins by SUMO1 was decreased by the K9R mutation in HEK293 cells. The presence (+) or absence (−) of mutations at K9 in each construct is indicated above the lane. A representative immunoblot result from three independent experiments is shown.

**Figure 2:**
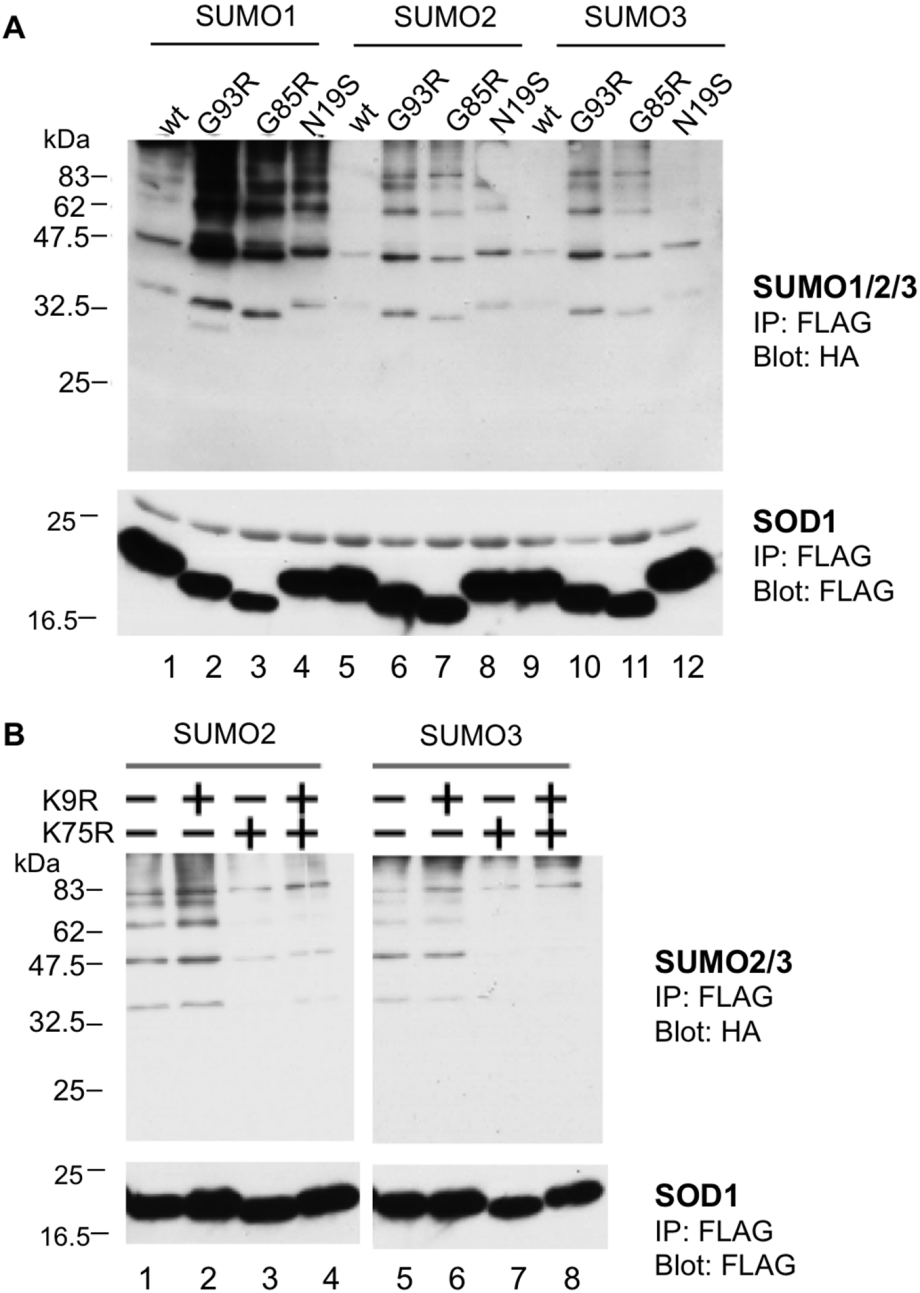
SOD1 proteins are modified by SUMO1, SUMO2, and SUMO3. NSC34 cells were cotransfected with plasmids expressing FLAG-tagged SOD1 (either wild-type or mutant), HA-tagged SUMO 1/2/3, and myc-tagged Ubc9. The cell lysates were immunoprecipitated with an anti-FLAG M2 antibody, followed by immunoblotting with anti-HA antibody (upper panels) and anti-FLAG antibody (lower panels). **A.** SOD1 mutants (G93R, G85R, and N19S) were modified by both SUMO1 and SUMO2/3. A representative immunoblot result from four independent experiments is shown. **B.** The sumoylation of G93R-SOD1 by SUMO2/3 was markedly decreased by the K75R mutation but not by the K9R mutation. The presence (+) or absence (−) of mutations at K9 and K75 is indicated above the lane. A representative immunoblot result from three independent experiments is shown.

To understand the role of sumoylation in the pathobiology of ALS, we used NSC34 cells, a motor neuron cell line [23] that is widely used for studies on the pathomechanisms of ALS. First, we examined whether the SOD1 protein undergoes sumoylation in these cells by cotransfecting FLAG-tagged wild-type (wt) or mutant SOD1 with HA-tagged SUMO1 in the presence of myc-tagged Ubc9, a sumoylation E2 conjugase. The FLAG-SOD1 proteins were immunoprecipitated with an anti-FLAG antibody, and the precipitates were subjected to western blotting using an anti-HA antibody to detect HA-SUMO1. Two dominant bands with molecular masses of approximately 38 and 58 kDa, corresponding to the size of putative mono- and di-sumoylated SOD1, respectively, were detected in cells expressing each SOD1 protein (Fig. 1A, Fig. 2A, lanes 1–4, and Fig. S1A, lanes 1 and 2, arrowheads). Minor bands with a higher molecular mass were also detected but were not derivatives of sumoylated SOD1 because the anti-FLAG antibody did not detect these bands in the anti-FLAG (Fig. S1A and B, lane 5) or anti-HA (Fig. S1A and B, lane 8) antibody precipitates, suggesting that sumoylated proteins other than SOD1 were also coimmunoprecipitated with FLAG-SOD1. These observations indicate that all the SOD1 proteins, including wt, FALS mutants, and N19S mutant, were modified by SUMO1 in the NSC34 cells. The degree of sumoylation was higher for the FALS mutant proteins (Fig. 1A, lanes 3–5, and Fig. 2A, lanes 2 and 3) than the wt (Fig. 1A, lane 2, and Fig. 2A, lane 1) and N19S mutant proteins (Fig. 1A, lane 6, and Fig. 2A, lane 4). The difference in SUMO1-modification between the FALS mutants and wt was not due to a difference in sumoylation E2 conjugase activity because the levels of Ubc9 were almost the same in both the lysates of cells transfected with the FALS mutants and wt SOD1 and because the global sumoylation of cellular proteins occurred similarly in both cells (Fig. S1C). The sumoylation of SOD1 appears to occur for a portion of SOD1 proteins, as a large amount of nonsumoylated monomer SOD1 proteins (Figs. S1A and B, lanes 4–6, indicated with an arrow) and small amount of nonsumoylated dimer proteins (Fig. S1A, lanes 4–6, Fig. S1D, indicated with asterisks) were detected in the anti-FLAG antibody immunoprecipitates.

There are two potential lysine residues for sumoylation, Lys9 and Lys75, in the SOD1 protein. To identify which lysine residue is important for SOD1 sumoylation, we substituted these lysine residues with an arginine. As shown in Figure 1B, both K9R and K75R mutations reduced the SUMO1 modification of G93R-SOD1 (Fig. 1B, lanes 2 and 3), and the degree of sumoylation was the lowest in G93R-SOD1 carrying two K/R mutations (Fig. 1B, lane 4). These results indicate that G93R-SOD1 can be sumoylated at both Lys9 and Lys75 in a cell line of neuronal origin. We also confirmed the site specificity of sumoylation on the SOD1 protein in HEK293 cells. The K9R mutations significantly reduced the sumoylation of all types of SOD1 proteins (Fig. 1C, lanes 2, 4, 6, and 8), indicating that SOD1 can also be modified by SUMO1 via Lys9 in HEK293 cells.

### SOD1 mutants are modified by SUMO2 and SUMO3 at Lys75

Fei *et al*. showed that wt SOD1 was not modified by SUMO2 or SUMO3 in HEK293 cells [22]. We therefore examined whether SOD1 is modified by SUMO2 and SUMO3 in neuronal cells by immunoprecipitating FLAG-SOD1 with an anti-FLAG antibody, followed by the detection of HA-SUMO with an anti-HA antibody. Consistent with the finding of Fei *et al*. [22], neither SUMO2 nor SUMO3 caused significant modification of wt SOD1 in NSC34 cells (Fig. 2A, lanes 5 and 9). However, the FALS-linked mutant SOD1 proteins G93R and G85R were clearly modified by SUMO2 and SUMO3 (Fig. 2A, lanes 6, 7, 10, and 11, and Fig. S1A, lane 3, arrowheads). Similar to the cells transfected with SUMO1, two bands with sizes corresponding to sumoylated FLAG-G93R-SOD1 protein were commonly detected in the anti-FLAG antibody (Figure S1A, lane 6, arrowheads) and anti-HA antibody (Fig. S1A, lane 9, arrowheads) immunoprecipitates, suggesting that these bands are mono- and di-sumoylated SOD1 proteins. The sumoylation of mutant SOD1 proteins was marginally detectable in the absence of exogenously transfected Ubc9 but was significantly increased by the overexpression of Ubc9 (Fig. S2A), indicating that the regular sumoylation machinery also functions in the SUMO modification of SOD1 mutants. As is the case with SUMO1, the difference in SUMO3 modification between the FALS mutants and wt was not due to a difference in the sumoylation E2 conjugase activity because the levels of Ubc9 and global sumoylation of cellular proteins were almost the same in the lysates of cells transfected with the FALS mutants and wt SOD1 (Fig. S1C).

In addition to the FALS-linked mutant SOD1 proteins, N19S-SOD1 was also modified by both SUMO2 and SUMO3 but at a lower degree compared to the FALS-linked SOD1 mutants (Fig. 2A, lanes 8 and 12). We next used the K/R mutants to determine the sumoylation sites in G93R-SOD1. The K75R mutation, but not the K9R mutation, significantly reduced G93R-SOD1 sumoylation by SUMO2 and SUMO3 (Fig. 2B, lanes 3 vs. 2, and 7 vs. 6), indicating that the SUMO2/3 modification of the mutant SOD1 proteins mainly occurs at Lys75.

### SUMO3 increases mutant SOD1 protein aggregation

**Figure 3:**
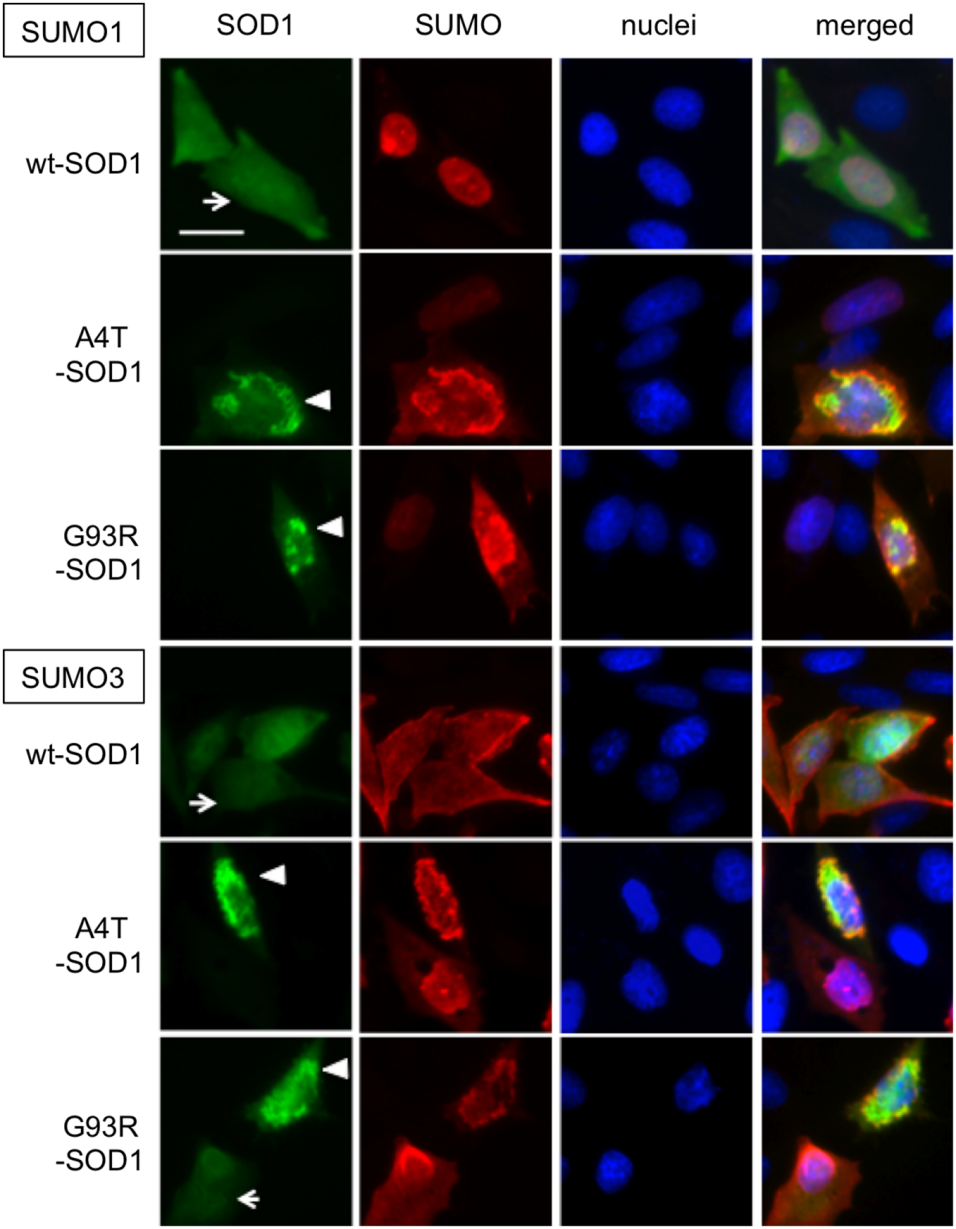
SUMOs colocalize with familial ALS-linked SOD1 mutants in intracellular aggregates. CHO cells were cotransfected with plasmids expressing EGFP-fused SOD1 (either wild-type or mutant), HA-tagged SUMO 1/3, and myc-tagged Ubc9. The cells were fixed after 24 h of transfection and immunostained with anti-HA antibody and DyLight594-conjugated anti-mouse IgG antibody, followed by counterstaining with DAPI. Representative images from two independent experiments are shown. The bar in the upper left panel indicates 10 µm. Arrowheads and arrows indicate cells with and without intracellular SOD1 protein aggregates, respectively. In all triple transfections, all GFP-positive cells were DyLight594 (SUMO)-positive (detail in Supporting Information Table S1).

**Figure 4:**
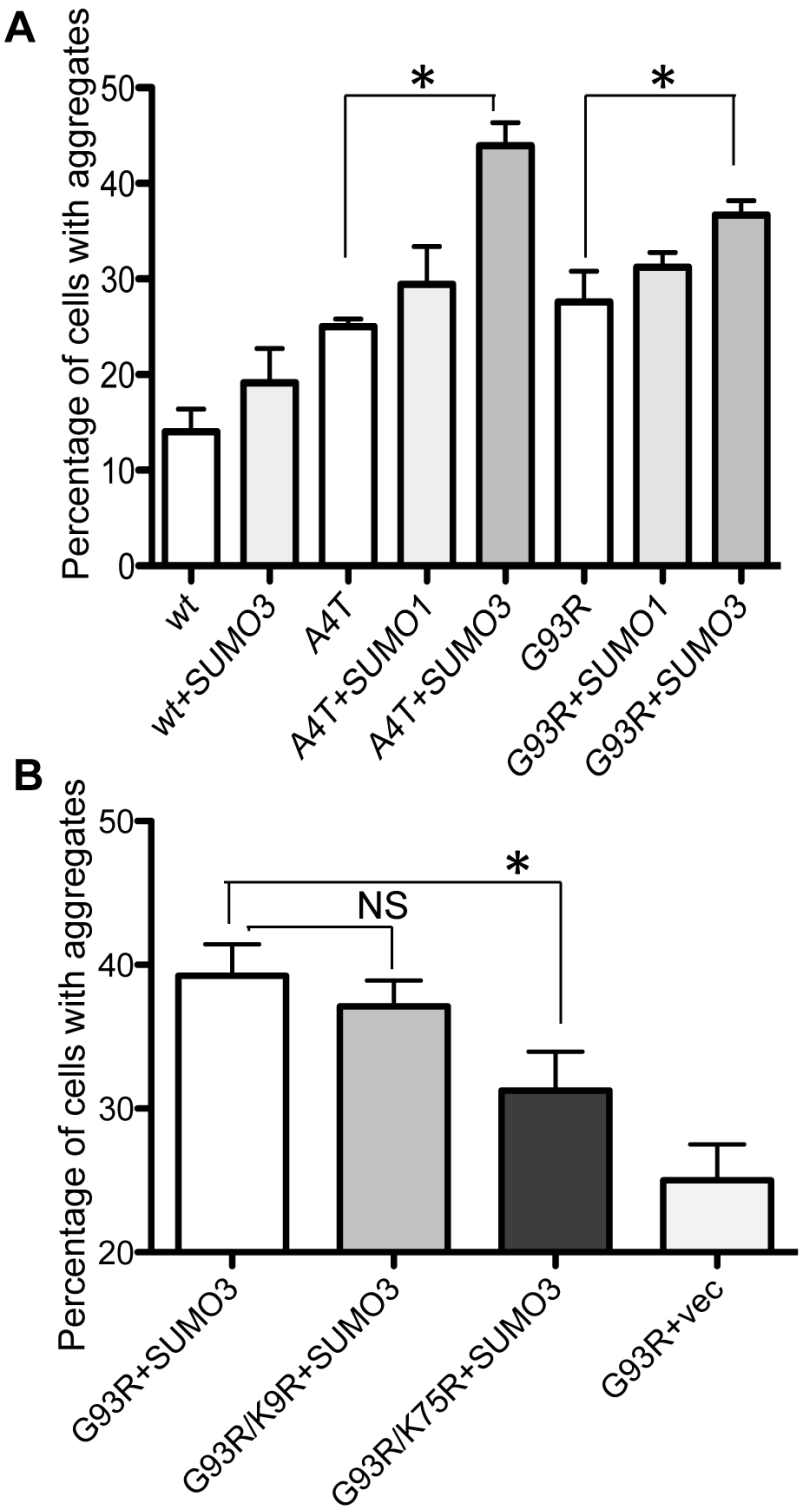
SUMO3 modification increases the aggregation of SOD1 proteins. CHO cells were cotransfected with plasmids expressing EGFP-fused SOD1 (either wild-type or mutant), HA-tagged SUMO 1/3, and myc-tagged Ubc9 and fixed after 24 h of transfection. Images of randomly selected fields in each well were recorded, and the number of cells with and without aggregates were counted. The total number of GFP-positive cells counted in each sample (mean ± SD) were 185±49 (A) and 338±124 (B). **A.** SUMO3 increased aggregate formation of mutant SOD1 proteins. The percentages of aggregate-positive cells (mean and SD) were plotted (triplicate sample). Statistically significant differences (p<0.05) between with and without SUMO3 were analyzed by t-test and indicated by asterisks. **B.** K75R mutation significantly reduced the aggregate formation of G93R-SOD1. The percentages of aggregate-positive cells (mean and SD) were plotted (sextuplicate samples). Statistically significant difference (p<0.05) by a one-way ANOVA followed by a post-hoc test is indicated by asterisks. N.S. indicates not significant.

We have previously demonstrated that FALS-linked SOD1 mutant proteins aggregate in CHO cells using EGFP-fused SOD1 proteins [24]. Using this system, we then examined the effects of SUMO1 and SUMO3 on the aggregation of FALS-linked mutant SOD1 proteins. First, we observed the subcellular localization of SUMO1 and SUMO3 cotransfected with wt-SOD1-EGFP by immunofluorescent staining using an anti-HA antibody (Fig. 3). As reported previously, SUMO1 and SUMO3 are mainly localized to the nucleus and cytosol, respectively. Regardless of the localization pattern of each SUMO protein, wt SOD1-EGFP was found to be localized throughout the cell body (Fig. 3 arrows). In contrast, FALS-mutant SOD1-EGFP formed aggregates in the perinuclear area (Fig. 3 arrow heads), and a portion of the SUMO protein colocalized with the aggregates. A quantitative analysis (Fig. 4A) showed that the coexpression of SUMO1 and Ubc9 with A4T/G93R-SOD1 slightly, but not significantly, increased the number of aggregate-positive cells, whereas significant increases in aggregate formation were observed in the cells coexpressing SUMO3, Ubc9, and A4T/G93R-SOD1. These results indicate that SUMO3 has more influence on the aggregate formation of mutant SOD1 than does SUMO1. To examine whether the enhancement of aggregation of G93R-SOD1 by coexpression of SUMO3 is induced by direct SUMO3 modification of the SOD1 protein, we introduced the K/R mutations in the G93R-SOD1-EGFP fusion construct (Fig. 4B). Consistent with Figure 2B, K75R mutant but not K9R mutant significantly reduced the rate of aggregate positive cells, suggesting that SUMO3 modification on Lys75 directly contributes to the aggregate formation of mutant SOD1 proteins.

### SUMO3 modification stabilizes FALS-linked SOD1 mutants

**Figure 5:**
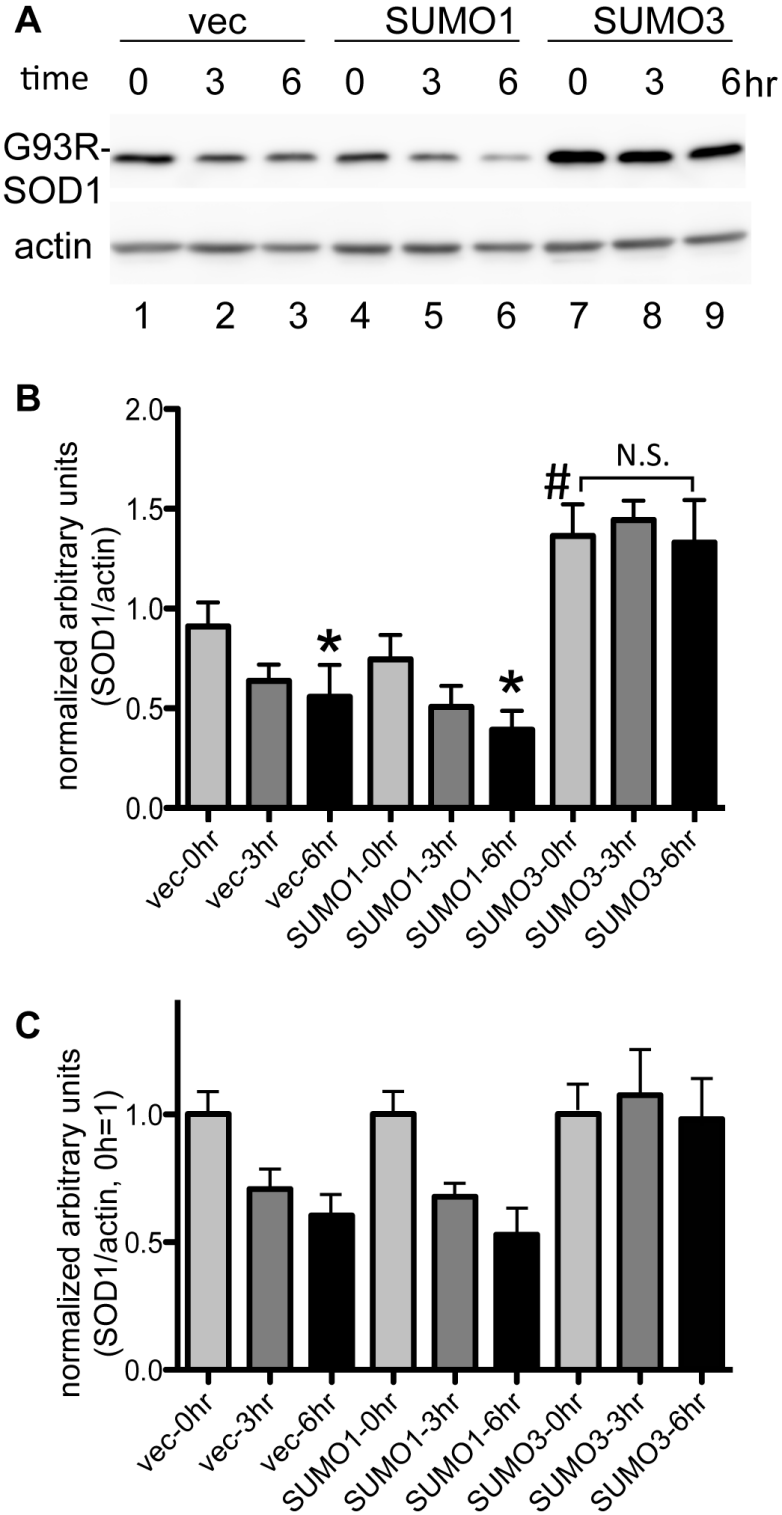
A familial ALS-linked SOD1 mutant is stabilized by SUMO3 modification. NSC34 cells were cotransfected with plasmids expressing FLAG-tagged G93R-SOD1, HA-tagged SUMO1, SUMO3, or the empty vector (vec), and myc-tagged Ubc9. After 16 h of transfection, the cells were treated with 50 µg/ml cycloheximide for 3 or 6 h or left untreated (time 0). The cell lysates were subjected to an immunoblot analysis with anti-FLAG antibody (**A** upper panel) and anti-β-actin antibody (**A** lower panel). **A.** A representative immunoblot result is shown. **B.** Quantitative analysis of the immunoblot. The intensity of each band was quantified using ImageJ and normalized to the arbitrary units of β-actin, and the means and SD (n = 4) were calculated. # indicates a statistically significant difference (p<0.01) among the three transfection conditions (vec, SUMO1, SUMO3) at time 0. Statistically significant differences (p<0.05) between time 0 of each condition are indicated by *. N.S. indicates not significant. **C.** Quantitative analysis of the immunoblot. The data in B are expressed relative to the 0 h value ( = 1).

We next examined whether sumoylation affects the stability of FALS-linked mutant SOD1 proteins in NSC34 cells (Fig. 5). NSC34 cells expressing G93R-SOD1, SUMO1/3, and Ubc9 were treated with cycloheximide (CHX), a protein synthesis inhibitor, and the expression levels of the G93R-SOD1 protein were analyzed along a time course. The G93R-SOD1 levels similarly decreased during CHX treatment in a time-dependent manner, with or without coexpresion of SUMO1 (Fig. 5B, C). On the other hand, the coexpression of SUMO3 significantly increased the initial G93R-SOD1 levels (Fig. 5A, lane 7, and 5B) compared to that of SUMO1 and, in contrast to the coexpression of SUMO1, maintained the high level of G93R-SOD1 up to 6 h of CHX treatment (Fig. 5A, lanes 8 and 9, and 5B, C). The cells expressing the A4T-SOD1 protein showed results similar to those expressing G93R-SOD1, i.e., SUMO3 itself significantly increased the level of A4T-SOD1 (Fig. S3A, lane 7, and Fig. S3B), and the level of the protein was maintained during 6 h of CHX treatment (Fig. S3A, lanes 8 and 9, and Fig. S3B, C). These results suggest that SUMO3 plays a pivotal role in stabilizing FALS-linked mutant SOD1 proteins. Interestingly, our immunoblotting results with the anti-FLAG antibody exhibited a band with a size of approximately 19 kD (Fig. S1A, lanes 8 and 9, arrow), corresponding to nonsumoylated SOD1, in the anti-HA antibody immunoprecipitate from the lysate of cells expressing HA-SUMO and G93R-SOD1-FLAG. However, this 19-kDa band was not detectable in the precipitates from cells expressing HA-SUMO and wt SOD1-FLAG (Fig. S1B, lanes 8 and 9, arrow). Considering the fact that the majority of the SOD1 protein was not sumoylated (Figs S1A and B, lanes 4–6, indicated with an arrow), these results strongly suggest that the SUMO conjugation of mutant SOD1 accelerates the aggregation of the protein, incorporating the nonsumoylated form.

## Discussion

In this study, we found that Lys75 of mutant SOD1 proteins is modified not only by SUMO1 but also by SUMO2 and SUMO3 (Fig. 2). Fei *et al*. showed that the SUMO1 modification of SOD1 increases its aggregation and stability [22], and we observed a similar phenomenon, though the effect was not prominent in motoneuronal NSC34 cells. In contrast, SUMO3 modification significantly accelerated intracellular aggregate formation and stabilized the FALS-linked SOD1 mutant proteins (Figs. 4, 5). Although SUMO1 and SUMO2/3 share the same process for conjugation at Lys residues of target proteins, each SUMO displays a preferential intracellular localization: SUMO1 mainly localizes to the nuclear membrane, and SUMO3 is localized in the cytosol (Fig. 3) [15], [25]. Because SOD1 is a cytoplasmic protein, SUMO3, rather than SUMO1, should be more readily accessible to SOD1. In addition, SUMO2 and SUMO3 are found in a free, nonconjugated form *in vivo*, whereas SUMO1 mainly exists in a conjugated form. SUMO2/3 conjugation is induced by cellular stimuli, such as heat-shock and oxidative stress [14]. Therefore, it is more likely that SOD1 is conjugated by SUMO2/3, rather than by SUMO1, under conditions of stress.

SUMO3 increased the stability of nonsumoylated SOD1 mutant proteins (Fig. 5). This finding raises the question of how SUMO3 increases the total amount of nonsumoylated SOD1 mutant protein. One possibility is that the elevated SUMO3 level induced the global stabilization of proteins via modification of proteasomal degradation system. We observed that the mutation at Lys75, the major sumoylation site of SUMO3, significantly reduced the aggregate formation of mutant SOD1 proteins (Fig. 4B), suggesting that SUMO3 modification directly affects the aggregate formation. Moreover, the nonsumoylated mutant SOD1 proteins coimmunoprecipitated with SUMOs, whereas the nonsumoylated wt SOD1 proteins, which do not form aggregates, were undetectable in the SUMO immunoprecipitates (Fig. S1). Therefore, it appears unlikely that the SUMO3-induced increase in mutant SOD1 aggregation is merely caused by increased levels of SOD1 proteins, though it cannot completely rule out the possibility that SUMO3-target proteins other than SOD1 are involved in aggregate formation of mutant SOD1 proteins. It is rather more likely that the global augmentation of SUMO3 modification increases the amount of sumoylated SOD1 proteins and causes the recruitment of nonsumoylated SOD1 mutant proteins into the intracellular aggregates formed by sumoylated SOD1 proteins, resulting in accelerated aggregation formation. In addition, it has been reported that mutant SOD1 can form prion-like aggregation and has spreading ability [26]. It is thus speculated that sumoylation may trigger such prion-like characteristics in mutant SOD1. Multiple lines of evidence indicate that SOD1 aggregate formation closely correlates with cytotoxicity in cellular models and decreased survival in model mice [1], [18]–[21], [23]. Taken together, it is assumed that the sumoylation-induced acceleration of SOD1 aggregation is linked to neurotoxicity in ALS pathogenesis.

We also demonstrated that SOD1 can be modified by SUMO1 at two sites, Lys9 and Lys75, in motoneuronal NSC34 cells (Fig. 1). Both Lys9 and Lys75 are located within a canonical consensus sequence for sumoylation, ψKxE/D (where ψ is an aliphatic amino acid): LKGD and PKDE, respectively. SUMO1 modification at Lys75, but not Lys9, was previously demonstrated by an *in vitro* assay using purified proteins [22]. However, the additional observed sumoylation at Lys9 is most likely because of the difference between the assay systems, i.e., *in vitro* versus *in vivo*. Consistent with our findings, proteomic analyses have demonstrated SOD1 sumoylation at multiple sites in a yeast system [27], [28]. It should be noted that sumoylated SOD1 was still observed in the lysate of cells transfected with G93R/K9R/K75R-SOD1 triple mutant and SUMO1 (Fig. 1B, lane 4), suggesting the existence of minor sumoylation sites other than Lys9 and Lys75 in human SOD1.

Oxidative stress results from an imbalance in the production and removal of reactive oxygen species. Because free radical production increases with aging, oxidative stress is a risk factor for the onset of aging-related neurodegenerative disorders, including ALS [29]. Indeed, elevated oxidative damage in proteins is observed in sporadic ALS patients [30], [31] and in an ALS model of SOD1 transgenic mice [32]. SUMO2/3 conjugation is a major response to oxidative stress [8], [33], [34], and global protein modification by SUMO2/3 is increased by hydrogen peroxide treatment in yeast [14] and HeLa cells [35], supporting the link between oxidative damage and SUMO2/3 protein modification. A cellular model of ischemia induced by oxygen and glucose deprivation also increases global protein modification by SUMO2/3. The stress-induced SUMO2/3 modification in this model appears to function as a neuroprotective mechanism because the knockdown of SUMO2/3 increases cellular vulnerability to ischemic stress [36], [37]. Thus, it is unclear whether oxidative stress-induced sumoylation is neurotoxic or neuroprotective. Nevertheless, we propose that the stress-induced elevation of global sumoylation accelerates the aggregation of mutant SOD1 proteins and consequently activates cytotoxic pathways in motor neurons.

DNA-binding protein 43 (TDP-43) has been identified as a major aggregating protein in ALS and frontotemporal lobar degeneration [38], and TDP-43-positive aggregates are found in most sporadic cases and in familial cases caused by its mutation [39]. TDP-43 has a canonical sumoylation motif at positions 135–138, colocalizes with SUMO2/3 in intracellular inclusions, and is directly sumoylated in insoluble cellular protein fractions [40]. Additionally, heat-shock induces the accumulation of SUMO2-conjugated TDP-43 by 7-fold [41]. These findings suggest that SUMO2/3 is involved in TDP-43 aggregation under stress conditions. Considering our results, we propose that stress-induced SUMO2/3 modification plays an important role in the aggregate formation of ALS-associated proteins, which contributes to the pathogenesis of ALS.

## Materials and Methods

### Plasmids

Plasmids containing wt, FALS-linked SOD1 mutants (G93R, G85R, and A4T), and N19S-SOD1 cDNA were previously described [24]. K9R and K75R mutations were introduced via site-directed mutagenesis using the primers 5′-GTGTGCGTGCTGAGGGGCGACGGCCCAG-3′ (K9R sense), 5′-CTGGGCCGTCGCCCCTCAGCACGCACAC-3′ (K9R antisense), 5′-CACGGTGGGCCAAGGGATGAAGAGAGGC-3′ (K75R sense), and 5′-GCCTCTCTTCATCCCTTGGCCCACCGTG-3′ (K75R antisense). The pXJ-HA-SUMO1 and pXJ-myc-Ubc9 plasmids were provided by Dr. Victor Yu (National University of Singapore, Singapore). The SUMO2 and SUMO3 cDNAs were cloned by PCR from the human brain cDNA library using the primers 5′-GGATCCATGGCCGACGAAAAGCCCAAG-3′ (SUMO2 sense), 5′-CCCGGGTCAGTAGACACCTCCCGTCTG-3′ (SUMO2 antisense), 5′-GGATCCATGTCCGAGGAGAAGCCCAAG-3′ (SUMO3 sense), and 5′-CCCGGGCTAGAAACTGTGCCCTGCCAG-3′ (SUMO3 antisense); the products were inserted into the pXJ-HA vector.

### Cell culture, transfection, and immunoprecipitation

HEK293 and NSC34 cells, the hybrid cells of motor neuron-enriched embryonic mouse spinal cord cell with mouse neuroblastoma N18TG2[23], were cultured in Dulbecco’s modified Eagle’s medium (D-MEM) supplemented with 10% fetal bovine serum (FBS), 50 units/ml of penicillin, and 50 µg/ml of streptomycin (Invitrogen, Carlsbad, CA, USA). HEK293 (7×10^5^ cells/dish) and NSC34 cells (5×10^5^ cells/dish) were seeded into 60-mm dishes and transfected with plasmids expressing FLAG-tagged SOD1 (wt or mutant), HA-tagged SUMO 1/2/3, and myc-tagged Ubc9 by lipofection (Lipofectamine with Plus reagent, Invitrogen). To prepare the transfection mixture, the plasmid DNAs were mixed in a ratio of 2∶1∶1 for SOD1:SUMO:Ubc9. After 24 h, the cells were harvested and lysed in RIPA buffer (10 mM sodium phosphate [pH 7.2], 150 mM NaCl, 1% Triton X-100, 0.1% SDS, 1% sodium deoxycholate, 10 mM N-ethylmaleimide, and protease inhibitors [Complete protease inhibitor cocktail, Roche Diagnostics, Indianapolis, IN, USA]. The cell debris was removed by centrifugation (16,000×g, 20 min, 4°C), and the clarified cell lysates (500 µg of protein) were subjected to immunoprecipitation using anti-FLAG M2 antibody-conjugated beads (Sigma, St. Louis, MO, USA).

### Immunoblot analysis

Immunoprecipitates were subjected to SDS-PAGE, followed by transfer to polyvinylidene difluoride membranes (Millipore, Billerica, MA, USA). The membranes were blocked with 10% skim milk in TBS-T (20 mM Tris-HCl [pH 7.6], 136 mM NaCl, and 0.1% Tween 20) and incubated with horseradish peroxidase (HRP) conjugated to various primary antibodies in 1% skimmed milk in TBS-T, followed by detection with an enhanced chemiluminescence reagent (Thermo Scientific, Waltham, MA, USA). The primary antibodies used were HRP-conjugated anti-HA (Roche), HRP-conjugated anti-FLAG (Sigma), and HRP-conjugated anti-actin (Sigma) antibodies.

### Protein stability analysis

NSC34 cells (1.5×10^5^ cells/well) were seeded into 12-well plates and transfected with one of the plasmids expressing FLAG-tagged A4T-SOD1, HA-tagged SUMO 1/3, or the empty vector and myc-tagged Ubc9 using lipofection. After 16 h of transfection, the cells were treated with 50 µg/ml cycloheximide (CHX) for 3 or 6 h and lysed in T-PER lysis buffer (Thermo Scientific) with phosphatase and protease inhibitors (Thermo Scientific). The cell debris was removed by centrifugation, and the clarified cell lysates (20 µg of protein) were subjected to an immunoblot analysis as described above. The band intensity was quantified using ImageJ (NIH), and the arbitrary units of SOD1 were normalized to those of β-actin.

### Immunocytostaining

CHO cells were cultured in Ham’s F12 supplemented with 10% FBS, 50 units/ml penicillin, and 50 µg/ml streptomycin. The cells (8×10^4^ cells/well) were seeded into 4-well chamber slides (Thermo Scientific) and cotransfected with plasmids expressing enhanced green fluorescent protein (EGFP)-fused SOD1 (wt or mutant), HA-tagged SUMO1/3, and myc-tagged Ubc9 by lipofection. To prepare the transfection mixture, the plasmid DNAs were mixed in a ratio of 2∶1∶1 for SOD1:SUMO1/3:Ubc9. After 24 h of transfection, the cells were fixed with 4% paraformaldehyde in PBS and incubated with blocking solution (5% bovine serum albumin in PBS), anti-HA antibody (M180-3, MBL, Nagoya, Japan), and DyLight594-conjugated anti-mouse IgG antibody (Jackson Immuno Research, West Grove, PA, USA), followed by counterstaining with 4′, 6-diamidino-2-phenylindole (DAPI, AAT Bioquest).

### Quantification of SOD1 aggregates

CHO cells (4×10^5^ cells/well) were seeded into 6-well plates and cotransfected with plasmids expressing EGFP-fused SOD1 (wt or mutant), HA-tagged SUMO1/3, and myc-tagged Ubc9 by lipofection. After 24 hours, the cells were fixed with 4% paraformaldehyde in PBS, and EGFP fluorescence was detected using a fluorescence microscope (Olympus). Images of randomly selected fields in each well were recorded. We counted cells containing heterologous accumulation of fluorescence with high intensity as aggregate-positive cells (indicated with arrowheads in Fig. 3), and the percentages of aggregate-positive cells were calculated.

### Statistical analysis

All statistical analyses were performed using Prism5 (Graph Pad Software, La Jolla, CA, USA). Two group comparison was analyzed by t-test. Group differences were analyzed by a one-way ANOVA, followed by post-hoc tests (Tukey’s multiple comparison test). The results expressed as mean ± SD.

## Supporting Information

Figure S1 SOD1 proteins are sumoylated by SUMO1 and SUMO3. NSC34 cells were cotransfected with plasmids expressing FLAG-tagged G93R- (**A**) or wild-type (**B**) SOD1, HA-tagged SUMO 1/3 or vector, and myc-tagged Ubc9. The cell lysates were immunoprecipitated with anti-FLAG M2 antibody or anti-HA antibody (M180-3, MBL). Immunoprecipitates (A, B) and input samples (15 µg protein) (C) were analyzed by immunoblotting with HRP-conjugated anti-HA, anti-FLAG, anti-myc, and anti-β-actin antibodies. In D, immunoprecipitate of anti-FLAG antibody from NSC34 cells expressing G93R-SOD1-FLAG, HA-SUMO3, and myc-Ubc9 (indicated as IP) and the lysate of HEK293 cells (15 µg protein) expressing G93R-SOD1-FLAG, HA-SUMO3, and myc-Ubc9 were analyzed side-by-side. Arrowheads indicate the bands commonly detected in immunoprecipitates of anti-FLAG and anti-HA antibodies, suggesting that these bands are sumoylated SOD1 proteins. Arrows indicate the bands of non-sumoylated SOD1 monomer. Non-sumoylated G93R-SOD1 but not wt SOD1 monomer was detected in immunoprecipitates of anti-HA antibody. Asterisks indicate the bands detected by anti-FLAG antibody in immnoprecipitates of anti-FLAG antibody and HEK293 lysate, but not in immnoprecipitate of anti-HA antibody, suggesting that these bands are nonsumoylated SOD1 dimers.
(TIF)

FIgure S2 Ubc9 promotes sumoylation of SOD1. NSC34 cells were cotransfected with plasmids expressing FLAG-tagged mutant SOD1, HA-tagged SUMO 1/3, and either myc-tagged Ubc9 or the empty vector. The presence (+) or absence (−) of Ubc9 is indicated above the lane. The cell lysates were immunoprecipitated with an anti-FLAG M2 antibody. Input samples (15 µg protein) (**A**) and immunoprecipitates (**B**) were analyzed by immunoblot with HRP-conjugated anti-HA, anti-FLAG, anti-myc, and anti-β-actin antibodies. The global sumoylation of cellular proteins was significantly increased in the presence of Ubc9 (**A** upper panel). Consistently, the amount of sumoylated SOD1 proteins was markedly increased in the presence of Ubc9 (**B** upper panel).
(TIF)

Figure S3 **A.** Familial ALS-linked SOD1 mutant is stabilized by SUMO3 modification. NSC34 cells were cotransfected with plasmids expressing FLAG-tagged A4T-SOD1, one of the HA-tagged SUMO1, SUMO3, or the empty vector, and myc-tagged Ubc9. After 16 h of transfection, the cells were treated with 50 µg/ml cycloheximide for 3 or 6 h or untreated (time 0). The cell lysates were subjected to an immunoblot analysis with anti-FLAG antibody (**A** upper panel) and anti-β-actin antibody (**A** lower panel). A representative immunoblot result is shown. **B.** Quantitative analysis of the immunoblot. The intensity of each band was quantified by using ImageJ and normalized to the arbitrary units of β-actin, and the means and SD (n = 3) were calculated. **C.** Quantitative analysis of the immunoblot. The data in B are expressed relative to the 0 h value ( = 1). Statistical analysis between 0 h and 6 h was performed by t-test and the p values are shown. The amount of SOD1 protein decreased over time in the presence of SUMO1. On the other hand, the amount of SOD1 proteins did not show an apparent difference among treatment time in the presence of SUMO3.
(TIF)

Table S1 Coexpression of SOD1 and SUMO in CHO cells. Transfection efficiency of SOD1 and SUMO in CHO cells is summarized (representative data of two independent experiments). Immunostained cells used in Figure 3 were counted. The numbers of DAPI-positive nuclei were used as total cell numbers. All GFP-positive cells were also DyLight-positive, indicating that SOD1 and SUMO were coexpressed in these cells.
(TIF)
