# Supplementary figures and images for: SUMO3 Modification Accelerates the Aggregation of ALS-Linked SOD1 Mutants
Source: PLoS One. 2014 Jun 27;9(6):e101080. doi: 10.1371/journal.pone.0101080 (PMC4074151; doi:10.1371/journal.pone.0101080)

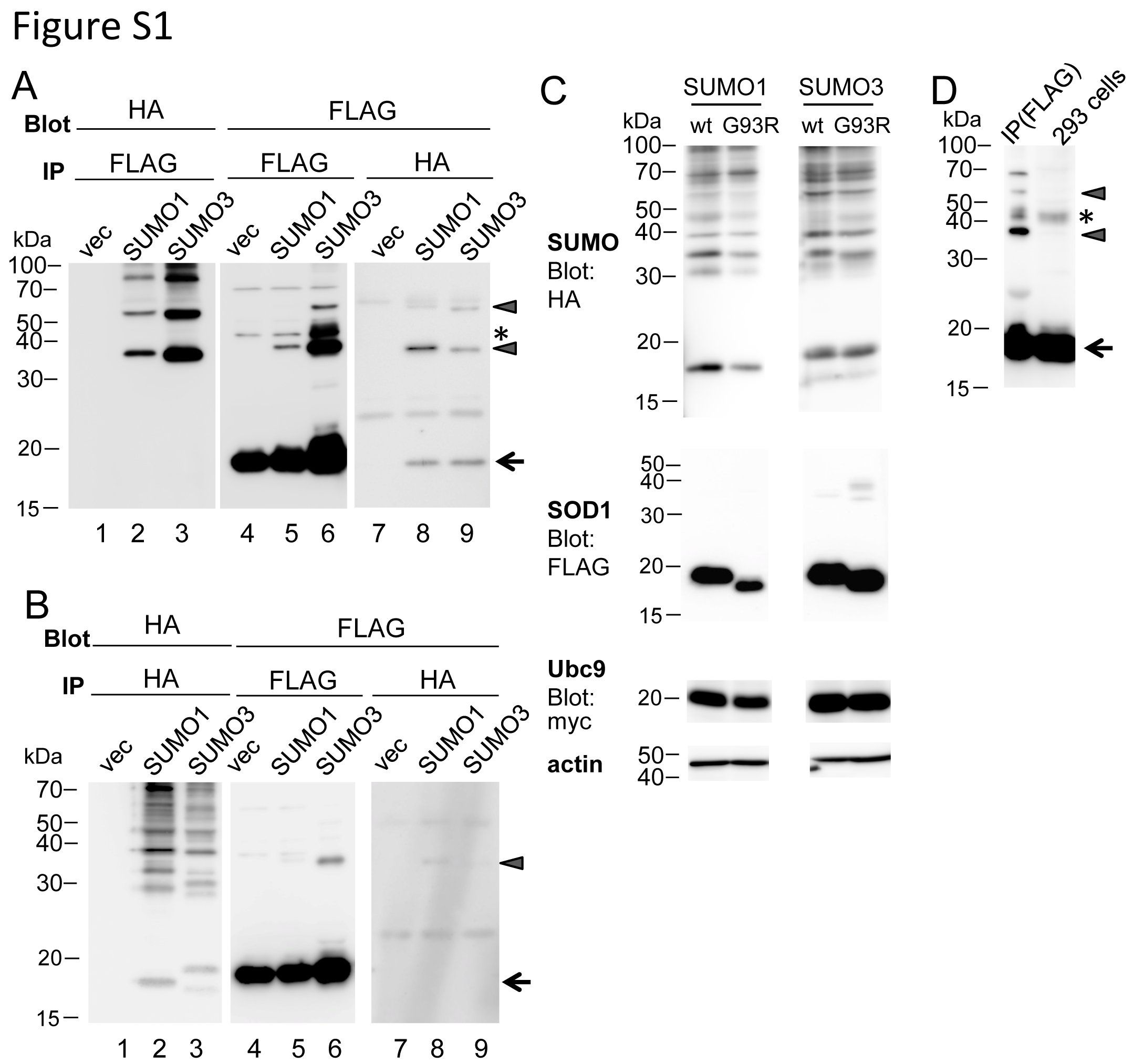

Supplement: Figure S1 — SOD1 proteins are sumoylated by SUMO1 and SUMO3. NSC34 cells were cotransfected with plasmids expressing FLAG-tagged G93R- (A) or wild-type (B) SOD1, HA-tagged SUMO 1/3 or vector, and myc-tagged Ubc9. The cell lysates were immunoprecipitated with anti-FLAG M2 antibody or anti-HA antibody (M180-3, MBL). Immunoprecipitates (A, B) and input samples (15 µg protein) (C) were analyzed by immunoblotting with HRP-conjugated anti-HA, anti-FLAG, anti-myc, and anti-β-actin antibodies. In D, immunoprecipitate of anti-FLAG antibody from NSC34 cells expressing G93R-SOD1-FLAG, HA-SUMO3, and myc-Ubc9 (indicated as IP) and the lysate of HEK293 cells (15 µg protein) expressing G93R-SOD1-FLAG, HA-SUMO3, and myc-Ubc9 were analyzed side-by-side. Arrowheads indicate the bands commonly detected in immunoprecipitates of anti-FLAG and anti-HA antibodies, suggesting that these bands are sumoylated SOD1 proteins. Arrows indicate the bands of non-sumoylated SOD1 monomer. Non-sumoylated G93R-SOD1 but not wt SOD1 monomer was detected in immunoprecipitates of anti-HA antibody. Asterisks indicate the bands detected by anti-FLAG antibody in immnoprecipitates of anti-FLAG antibody and HEK293 lysate, but not in immnoprecipitate of anti-HA antibody, suggesting that these bands are nonsumoylated SOD1 dimers. (TIF) [file pone.0101080.s001.tif]

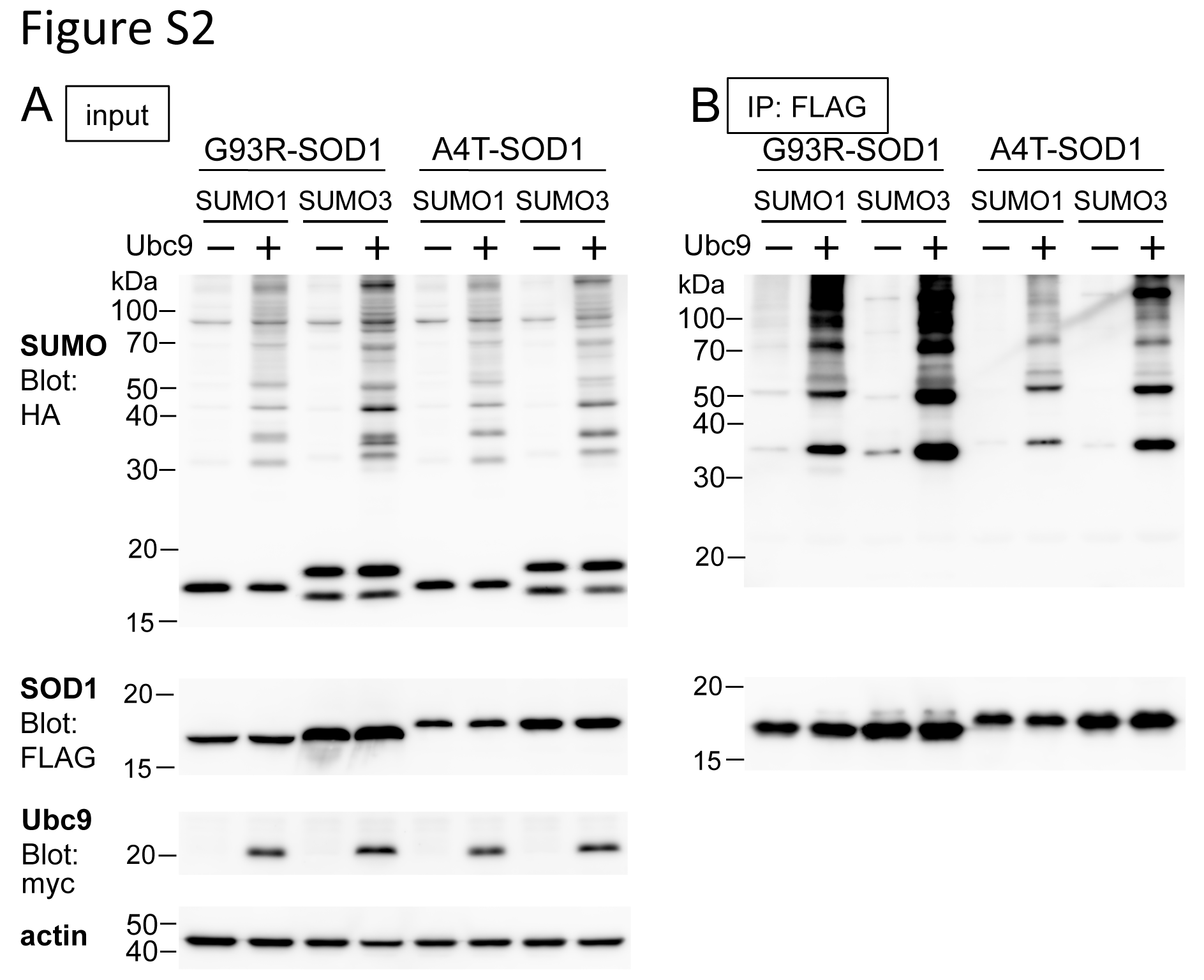

Supplement: FIgure S2 — Ubc9 promotes sumoylation of SOD1. NSC34 cells were cotransfected with plasmids expressing FLAG-tagged mutant SOD1, HA-tagged SUMO 1/3, and either myc-tagged Ubc9 or the empty vector. The presence (+) or absence (−) of Ubc9 is indicated above the lane. The cell lysates were immunoprecipitated with an anti-FLAG M2 antibody. Input samples (15 µg protein) (A) and immunoprecipitates (B) were analyzed by immunoblot with HRP-conjugated anti-HA, anti-FLAG, anti-myc, and anti-β-actin antibodies. The global sumoylation of cellular proteins was significantly increased in the presence of Ubc9 (A upper panel). Consistently, the amount of sumoylated SOD1 proteins was markedly increased in the presence of Ubc9 (B upper panel). (TIF) [file pone.0101080.s002.tif]

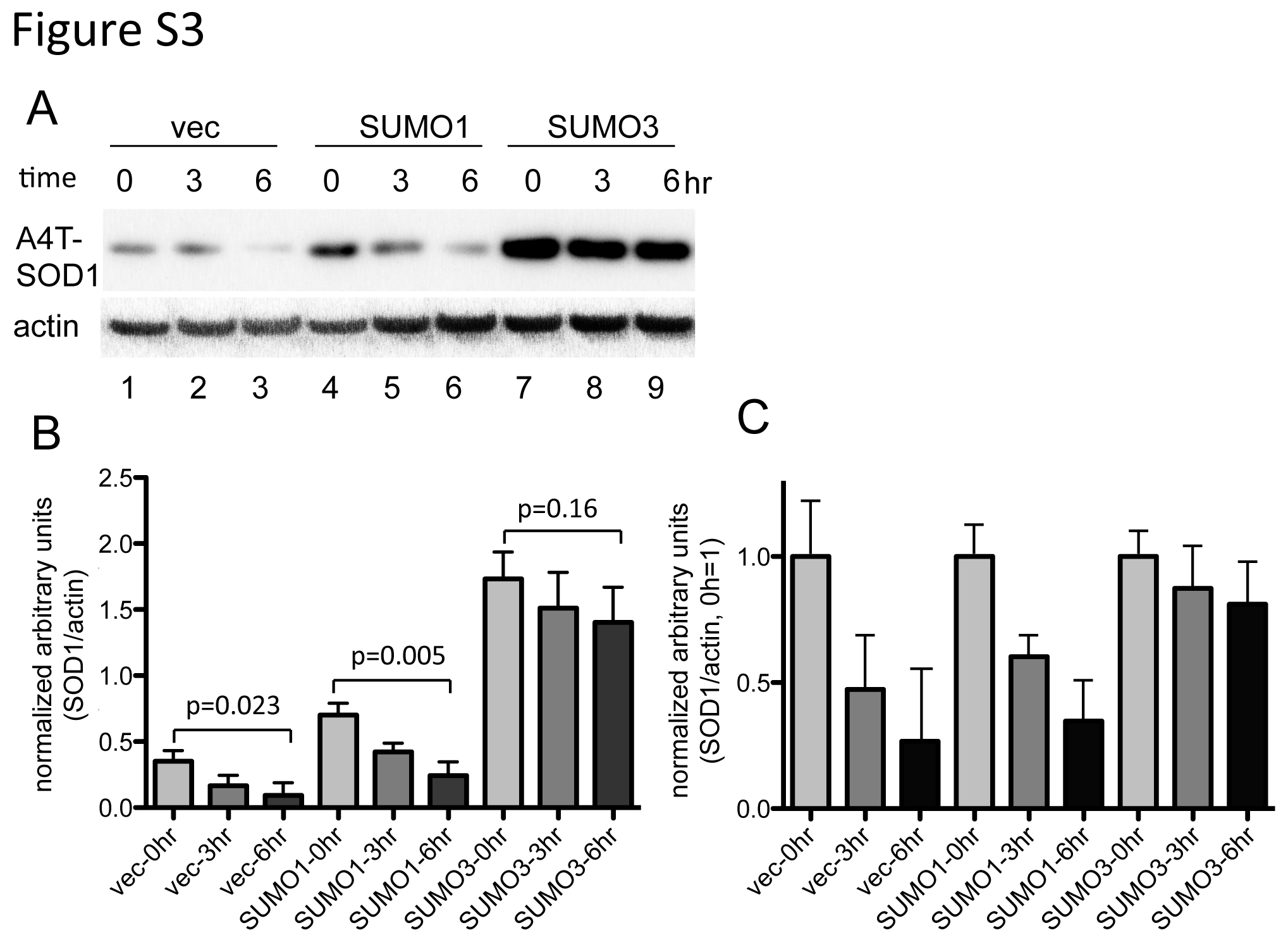

Supplement: Figure S3 — A. Familial ALS-linked SOD1 mutant is stabilized by SUMO3 modification. NSC34 cells were cotransfected with plasmids expressing FLAG-tagged A4T-SOD1, one of the HA-tagged SUMO1, SUMO3, or the empty vector, and myc-tagged Ubc9. After 16 h of transfection, the cells were treated with 50 µg/ml cycloheximide for 3 or 6 h or untreated (time 0). The cell lysates were subjected to an immunoblot analysis with anti-FLAG antibody (A upper panel) and anti-β-actin antibody (A lower panel). A representative immunoblot result is shown. B. Quantitative analysis of the immunoblot. The intensity of each band was quantified by using ImageJ and normalized to the arbitrary units of β-actin, and the means and SD (n = 3) were calculated. C. Quantitative analysis of the immunoblot. The data in B are expressed relative to the 0 h value ( = 1). Statistical analysis between 0 h and 6 h was performed by t-test and the p values are shown. The amount of SOD1 protein decreased over time in the presence of SUMO1. On the other hand, the amount of SOD1 proteins did not show an apparent difference among treatment time in the presence of SUMO3. (TIF) [file pone.0101080.s003.tif]

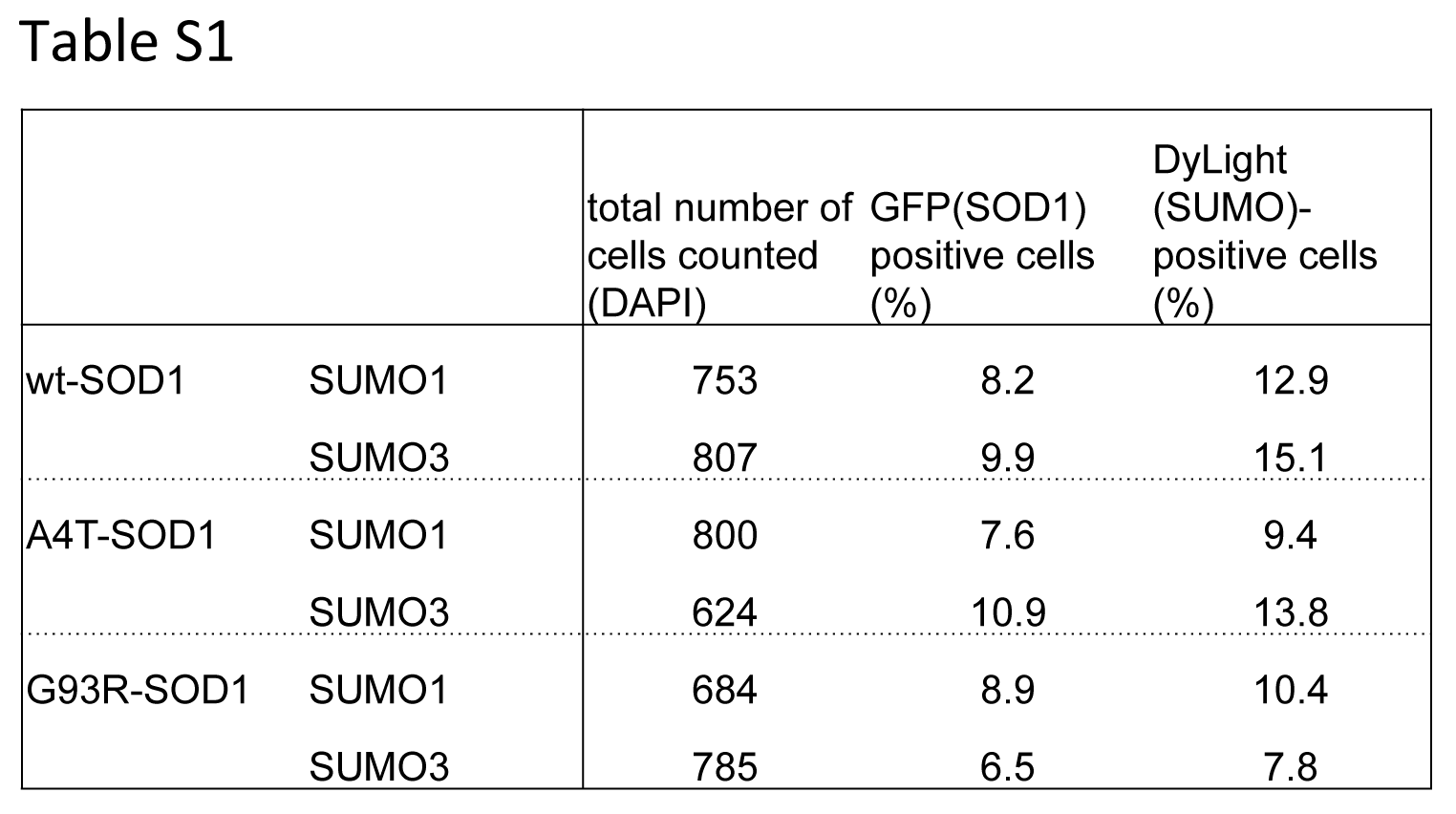

Supplement: Table S1 — Coexpression of SOD1 and SUMO in CHO cells. Transfection efficiency of SOD1 and SUMO in CHO cells is summarized (representative data of two independent experiments). Immunostained cells used in Figure 3 were counted. The numbers of DAPI-positive nuclei were used as total cell numbers. All GFP-positive cells were also DyLight-positive, indicating that SOD1 and SUMO were coexpressed in these cells. (TIF) [file pone.0101080.s004.tif]
